# Tularemia – a re-emerging disease with growing concern

**DOI:** 10.1080/01652176.2023.2277753

**Published:** 2023-11-18

**Authors:** Rinku Sharma, Rajendra Damu Patil, Birbal Singh, Sandip Chakraborty, Deepak Chandran, Kuldeep Dhama, Devi Gopinath, Gauri Jairath, Ajayta Rialch, Gorakh Mal, Putan Singh, Wanpen Chaicumpa, G. Saikumar

**Affiliations:** a Disease Investigation Laboratory, ICAR-Indian Veterinary Research Institute, Regional Station, Palampur, Himachal Pradesh, India; b Department of Veterinary Pathology, DGCN College of Veterinary and Animal Sciences, CSK HPKV, Palampur, Himachal Pradesh, India; c Department of Veterinary Microbiology, College of Veterinary Sciences and Animal Husbandry, R.K. Nagar, West Tripura, India; d Department of Animal Husbandry, Palakkad, Kerala, India; e Division of Pathology, ICAR-Indian Veterinary Research Institute, Izatnagar, Bareilly, Uttar Pradesh, India; f Center of Research Excellence in Therapeutic Proteins and Antibody Engineering, Department of Parasitology, Faculty of Medicine Siriraj Hospital, Mahidol University, Bangkok, Thailand

**Keywords:** Franscisella tularensis, zoonosis, bioterrorism agent, tularemia, rabbit fever, lymphadenopathy

## Abstract

Tularemia caused by Gram-negative, coccobacillus bacterium, *Francisella tularensis,* is a highly infectious zoonotic disease. Human cases have been reported mainly from the United States, Nordic countries like Sweden and Finland, and some European and Asian countries. Naturally, the disease occurs in several vertebrates, particularly lagomorphs. Type A (subspecies *tularensis*) is more virulent and causes disease mainly in North America; type B (subspecies *holarctica*) is widespread, while subspecies *mediasiatica* is present in central Asia. *F. tularensis* is a possible bioweapon due to its lethality, low infectious dosage, and aerosol transmission. Small mammals like rabbits, hares, and muskrats are primary sources of human infections, but true reservoir of *F. tularensis* is unknown. Vector-borne tularemia primarily involves ticks and mosquitoes. The bacterial subspecies involved and mode of transmission determine the clinical picture. Early signs are flu-like illnesses that may evolve into different clinical forms of tularemia that may or may not include lymphadenopathy. Ulcero-glandular and glandular forms are acquired by arthropod bite or handling of infected animals, oculo-glandular form as a result of conjunctival infection, and oro-pharyngeal form by intake of contaminated food or water. Pulmonary form appears after inhalation of bacteria. Typhoidal form may occur after infection *via* different routes. Human-to-human transmission has not been known. Diagnosis can be achieved by serology, bacterial culture, and molecular methods. Treatment for tularemia typically entails use of quinolones, tetracyclines, or aminoglycosides. Preventive measures are necessary to avoid infection although difficult to implement. Research is underway for the development of effective live attenuated and subunit vaccines.

## 1. Introduction

Tularemia, also known as “Rabbit fever, water-rat trappers’ disease, wild hare disease (yato-byo), and Ohara’s disease” (Stidham et al. 2018) is a rare but highly contagious zoonotic disease caused by Gram-negative, intracellular coccobacillus bacterium named *Francisella tularensis*. The number of host species susceptible to infection by this agent (>100 species) is higher than for any other known zoonotic pathogens (Hopla and Hopla 1994). Lagomorphs and rodents are the primary sources of human infections, but many other vertebrate species can be infected by *F. tularensis*. Human infections occur through several modes of infection like handling of infected animals, tick and mosquito bites, conjunctiva, contaminated food and water, and inhalation. Infection in human occurs as a result of contact with animals that are infected or the invertebrate vector of the disease (Auwaerter and Penn 2020). It was reported for the first time by McCoy and Chapin in 1912 as a disease comparable to plague in ground squirrel (*Citellus beecheyi*) in Tulare County, California (Mandell and Bennett 2005). Later, Edward Francis found that several clinical syndromes in humans were caused by *F. tularensis* and proposed the name ‘tularemia’ to describe the illness. In 1919, he also isolated the bacteria from a patient with deer fly fever, thereby demonstrating its transmission by arthropods (Francis 1925). *Francisella tularensis* is considered a life-threatening potential biological warfare agent and is classified as a Category A bioweapon by the Centre for Disease Control and Prevention (CDC) (Maurin 2015). It is one of the six agents listed in Tier 1 by the US Department of Health and Human Services (CDC USA website 2023). In Europe, *F. tularensis* is now being regarded as a re-emerging pathogen (Faber et al. 2018; Janse et al. 2018; Seiwald et al. 2020, Yeni et al. 2021). Although most tularemia cases can be treated with antibiotics (Dennis et al. 2001; Lindgren and Sjostedt 2016), it is still considered a life-threatening disease due to its high virulence (Su et al. 2007). The *F. tularensis* subspecies *tularensis* and subspecies *holarctica* are the only subspecies that cause tularemia in humans. The subspecies *mediasiatica* has never been isolated from humans but has been isolated from animals in central Asian countries. The *F. novicida* reported globally is considered a separate species (Johansson et al. 2000). It does not cause tularemia and is regarded as an opportunist pathogen (Eliasson et al. 2006).

## 2. Etiological agent and vectors

Tularemia is caused by *F. tularensis* which is a Gram-negative, catalase-positive, pleiomorphic, and non-motile coccobacilli belonging to the family Francisellaceae, order Thiotrichales, and class Gammaproteobacteria. It is a facultative, intracellular pathogen that can grow within different types of cells including macrophages, hepatocytes, and epithelial cells (Jia et al. 2009). Extraordinarily high level of fatty acids is present in the cell wall of *F. tularensis,* and the wild strains possess high fat-containing capsule, which has neither toxic nor immunogenic properties. It has been suggested that decapsulation decreases virulence, while the sustainability of the bacterium within neutrophils may remain unchanged (Nano 1998; Petersen et al. 2009). *Francisella tularensis* is a highly virulent pathogen and as few as ten living cells can cause potentially fatal disease in man and animals (Carvalho et al. 2014).

*Francisella tularensis* is divided into three subspecies, *tularensis*, *holarctica*, and *mediasiatica,* with different pathogenicity and geographic distributions (Olsufiev et al. 1959). The *F. tularensis* subspecies *tularensis* is regarded as the most virulent subspecies and is classified as type A. Two distinct genetic sub-populations identified as AI and AII, have different geographic distributions, hosts, and vectors. Sub-population AI has been further subdivided into groups AIa and AIb. Sub-population AII is found primarily in the western United States, whereas sub-population AI is found throughout the central and eastern regions of the country and sporadically in some western states. The subspecies *holarctica*, isolated from mild tularemia form, is classified as type B and reported from both the northern and southern hemispheres (Eden et al. 2017; Faber et al. 2018; Appelt et al. 2020). Infection with AIb strains in humans causes severe clinical disease and high mortality rates as compared to infections by AIa and AII strains or type B tularemia (Carvalho et al. 2014). In a study by Kugeler et al. (2009), significantly higher fatality rates were observed in patients infected with AIb strains as compared to those infected with AIa or AII strains. The higher mortality rate for AIb infection was not associated with host factors (age, sex, and underlying illness), indicating an intrinsic virulent characteristic of AIb strains. The virulence of subspecies *mediasiatica* is comparable to subspecies *holarctica*, but it has been reported from Central Asia to date (Carvalho et al. 2014). It is important to note that no human cases of infection have been described with the subspecies *mediasiatica* (Timofeev et al. 2020). About the fourth subspecies, there are variable reports. Some authors consider *novicida* to be a subspecies of *F. tularensis* (Fooladfara and Moradi 2023) while others consider *F. novicida,* as a separate species that is the least virulent (and opportunistic) (Johansson et al. 2000; Foley and Nieto 2010).

## 3. Reservoirs and vectors of *F. tularensis*

The *F. tularensis* subspecies *tularensis* was found in rabbits (*Sylvilagus* spp.) and hares (*Lepus* spp.), while those of *F. tularensis* subspecies *holarctica* were found in voles (*Microtus* spp.) and water voles (*Arvicola terrestris*) (Rijks et al. 2022). In mammals, tularemia is transmitted by arthropods like ticks, mosquitoes, and rarely deer flies (Petersen et al. 2009). The arthropod vectors associated with the disease include ticks of the genera *Amblyomma*, *Dermacentor*, *Ixodes*, and *Ornithodoros*; mosquitoes of the genera *Aedes*, *Culex*, *Anopheles*, and *Ochlerotatus excrucians*; and flies from the family Tabanidae (*Tabanus* spp., *Chrisozona* spp., and *Chrisops* spp.) (Ellis et al. 2002; World Health Organization (WHO)) 2007; Petersen et al. 2009; Dryselius et al. 2019). However, vector competence has only been demonstrated in ticks of the genera *Dermacentor* (Reese et al. 2010; Fooladfar and Moradi 2023). Interestingly, ticks act as both reservoirs as well as vectors of infection since they can carry the bacteria by transstadial as well as transovarial transmission. In Europe and most parts of America, the main source of the disease are ticks like *Ixodes ricinus* and *Dermacanter reticularis* (Petersen et al. 2009). In Sweden and Finland, most of the cases of tularemia occur by the bite of mosquitoes. In Sweden for the first time, it was reported that *Aedes cinereus* mosquitoes could be naturally infected by the bacterium (Olin 1941).

## 4. Geographical distribution

Type A tularemia is restricted to North America where type B is also encountered (World Health Organization (WHO)) 2007). The less virulent subspecies *holarctica* is predominantly found in Europe and Asia, usually triggering a subclinical or mild disease. The subspecies *holarctica* has also been detected in Tasmania, Australia (Carvalho et al. 2014). In the middle east, many people died from the disease, particularly in the 1400s BC (Trevisanato 2004). In Norway in the sixteenth century, tularemia was described for the first time as a disease of the rodent group, called lemming. In the 1800s, it was described in Russia as well as Japan (Gürcan 2014). Tularemia is considered a re-emerging disease in Europe (Bahuaud et al. 2021) with a four-fold increase in Switzerland and a 10-fold increase in Sweden in the last three decades (Imbimbo et al. 2020). A higher number of cases are reported annually from Scandinavian countries, like Finland and Sweden as compared to central and southern Europe (France, Hungary, Austria, Czech Republic, Germany, and Spain) (World Health Organization (WHO)) 2007). Since 2006, a small but steady increase in the number of tularemia cases has been reported in Poland. The early twenty first century witnessed the re-emergence of tularemia in north-western parts of Turkey, Bulgaria, Kosovo, and Georgia (Kilic 2010). Besides, sporadic case notifications have occurred in Estonia, Italy, Lithuania, Romania, Slovakia, Denmark, and the United Kingdom. In Portugal, the blood of an asymptomatic man and tick, *Dermacentor reticulatus* revealed the presence of *Francisella* by molecular techniques (Carvalho et al. 2014). In Germany, a total of 257 cases clinically manifested as glandular and ulcero-glandular tularemia were reported between 2002-2016 (Faber et al. 2018). Norway reported very high cases of tularaemia with oro-pharyngeal and ulcero-glandular forms being the most common (Kravdal et al. 2021). With the impact of climate change that has helped in the vector flourishes and persistence, the occurrence of tularemia amongst different animal species has increased (Bahuaud et al. 2021). In Asia, tularemia is endemic in Turkey, Azerbaijan, and Iran (Fooladfar and Moradi 2023). The disease has also been reported in Israel, Mongolia, China, and Japan (Eliasson et al. 2006).

In North America, *Francisella tularensis* subspecies *tularensis* is the most virulent type causing severe tularemia with a higher mortality rate, compared to subspecies *holarctica*. Except for Hawaii, tularemia has been reported in all states of the United States. In 2018, the overall incidence was 0.07 per 100,000 inhabitants; Arkansas, Oklahoma, Kansas, Missouri, and South Dakota are hotspots.

## 5. Host range

*Francisella tularensis* has been detected naturally in a high number of wild species including lagomorphs, rodents, insectivores, carnivores, ungulates, marsupials, birds, amphibians, fish, and invertebrates (Carvalho et al. 2014). Animals of orders Lagomorpha, Rodentia, and Sciuromorpha are thought to be the most important vertebrates that spread *F. tularensis*. There are a wide variety of rodent hosts, including voles, field mice, squirrels, lemmings, lagomorphs (cottontail rabbits), hares, jackrabbits, muskrats, and beavers (Kervyn et al. 2019; Kaya and Üçer 2022). Sheep, cats, rabbits, dogs, pigs, and horses are all susceptible to tularemia. Wild lagomorphs, such as the European brown hare (*Lepus europaeus*), are thought to be suitable sentinels for *F. tularensis* and disease surveillance. Birds are thought to be resistant to tularemia, although several species have developed a natural infection (Rijks et al. 2022; Sullivan et al. 2022). Lately, serological reports have indicated that foxes and raccoon dogs could also act as biological indicators for tularemia (Carvalho et al. 2014). Spontaneous infections with *F. tularensis* have also been recorded in different arthropods, though only a subset of these have been identified as important vectors in the transmission of the bacteria to human beings (Carvalho et al. 2014). There is a strong connection between the *F. tularensis* subspecies *holarctica* with the environment of fresh water, free-living amoeba, and biofilms. The mosquitoes’ larvae can be infected with *F. tularensis* since they can feed on the protozoa carrying the bacteria in the aquatic environment (Lundstrom et al. 2011; Ozanic et al. 2015).

## 6. Sources and mode of infection in animals

Transmission from animal to animal may occur through cutaneous, respiratory, or gastrointestinal routes. Transmission also occurs by blood-sucking arthropods including mites, ticks, flies, midges, fleas, mosquitoes, and lice. Transmission by these arthropods may be either mechanical by contaminated mouth parts or biological with the organism proliferating within the vectors and transmitted by bite or contamination of the host’s skin by excreta. The bacterium persists in the vector through transstadial transmission, even though the infected nymph ticks suffer high mortality due to the pathogen. Transmission by the transovarian route has also been described (Reese et al. 2011). Since tularemia is a septicemic disease and the affected animal is often depressed and lethargic, transmission by blood-sucking arthropods is greatly facilitated. Transmission may also occur by ingestion. Ingestion of infected carcasses may affect the spread of tularemia from animal to animal. Contamination of water of streams with *F. tularensis* from carcasses of infected rodents may take place. Ingestion of water by other vertebrates may transmit the disease. Transmission may occur by inhalation of feces, contaminated dust, or by inhalation of organisms in aerosols, leading to pneumonic tularemia (Seiwald et al. 2020). In the United States, deer flies and horse flies, and in Northern Eurasia mosquitoes play a crucial role in transmission of the organism. Rodents found in association with water, viz., ground as well as water voles, rats, raccoons, squirrels, and wild rabbits play an important role as reservoirs of infection in the natural cycle of *F. tularensis* subspecies *holarctica* (Geyik and Akalın 2009; Wobeser et al. 2009; Gürcan 2014).

## 7. Transmission to humans

**Figure 1.**
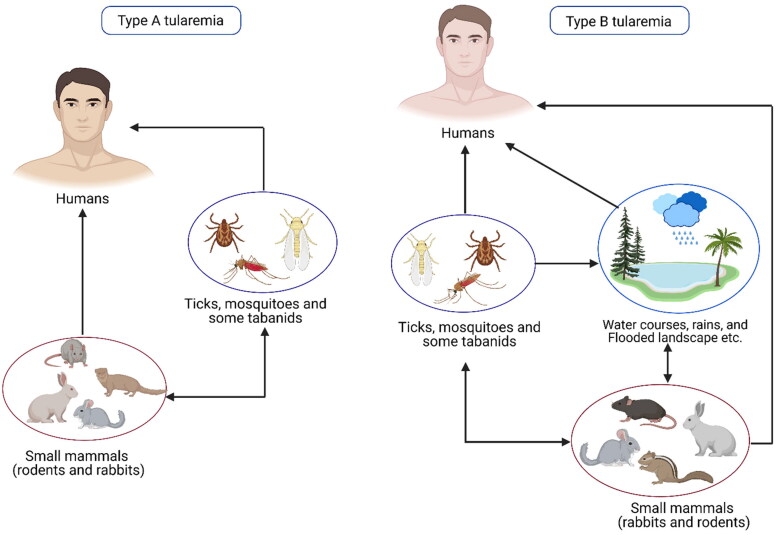
Modes of transmission of tularemia.

A pictorial representation of the modes of transmission of tularemia is shown in Figure 1. Many human infections occur after contact with a contaminated hydrotelluric environment (for example, skin injury in contact with vegetation or contaminated soil, swimming in contaminated water, etc.) (Magnarelli et al. 2007; Kleo et al. 2012). Transmission by direct contact with infected vertebrates is the most common mechanism whereby man is infected. Human cases in the United States are most frequently transmitted by tick bites (CDC USA website 2023). Human-to-human transmission has not yet been reported (Celli and Zahrt 2013; Carvalho et al. 2014).

Arthropod-borne tularemia outbreaks occur in Sweden and Finland due to transmission of the disease by mosquitoes. Ticks are thought to be the most important vectors of tularemia in the majority of countries where the disease is endemic. Both the ticks and mosquitoes may be infected in their larval phase. Tabanid flies are regarded as mechanical vectors for *F. tularensis*. However, long-term survival of this bacterium does not occur in these arthropods (Carvalho et al. 2014). Humans can contract *F. tularensis* by direct skin-to-skin contact with infected or living animal tissue, skin, or blood. People can become sick if they ingest uncooked animal tissues or meals or drink water tainted by infected carcasses or excrement (Parhizhgari et al. 2021). Human *F. tularensis* infections have been associated with submersion in water on occasion. Some agricultural jobs, like stacking hay or mowing lawns, put workers at risk for respiratory illnesses, particularly if they involve running over dead animals (Golovliov et al. 2021; Wawszczak et al. 2022).

In the United States, Sweden, Finland and Russia, the arthropod bite is a common mode of *F.* tularensis transmission to humans, whilst in Western and Central Europe, contact with infected animals and the ingestion of contaminated food or water have been reported as more common transmission modes. In Turkey, people are primarily infected by the consumption of contaminated spring water. These infections occur as outbreaks with a predominance of the oropharyngeal form (Kutlu et al. 2021). Recently, water-borne outbreaks of tularemia have also been reported in many European countries (Hennebique et al. 2019). Differences in transmission patterns have also been recorded within the United States, i.e. in Utah in the west, deer flies have been implicated in the transmission of tularemia in humans, while in other states, ticks are considered relevant.

Domestic dogs and cats can also transmit tularemia to humans after contact with an infected animal, environment, or infected ticks. A review of 10 years’ period in the United States revealed 3.3% of cases of human tularemia (1814 cases) were transmitted from dogs by numerous routes like direct contact *via* bite, scratch, or face snuggling/licking; direct contact with dead animals retrieved by domestic dogs; and contact with infected ticks acquired from domestic dogs (Kwit et al. 2019).

**Figure 2.**
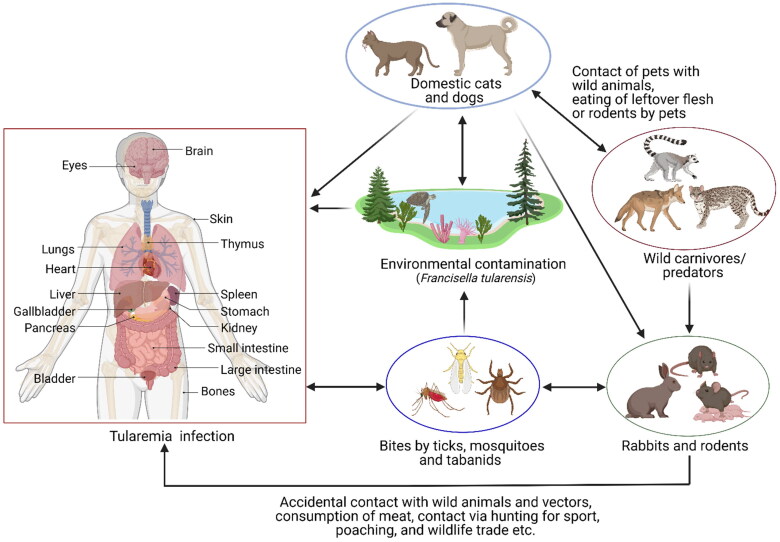
Diagrammatic representation of the terrestrial and aquatic cycles of tularemia.

The transmission cycle of *F. tularensis* shows deviations in different ecosystems. Both *F. tularensis* types A and B are associated with diverse life cycles in different animal hosts and arthropod vectors and can also co-exist (Figure 2). Type A tularemia is usually associated with the terrestrial cycle of the disease, with wild lagomorphs such as rabbits and hares being vertebrate hosts in which amplification of the agent occurs and where arthropods are disease-disseminating vectors. Type B tularemia is more frequently associated with the aquatic cycle, although outbreaks of tick-borne tularemia involving subspecies *holarctica* have been reported. In this life cycle, *F. tularensis* circulates in rodents including beavers, muskrats, and voles, and can be led into watercourses from animal carcasses. There is also evidence that *F. tularensis* can persist in watercourses in association with amebae. Contaminated water can be a source of infection to humans, flies, and mosquitoes. An unusual water-borne outbreak of human tularemia has been described in Spain associated with crayfish (*Procambarus clarkii*) caught in a contaminated freshwater stream. The *F. tularensis* was isolated from the stomach, liver, and pancreas of crayfish suggesting its role as a host (Carvalho et al. 2014).

## 8. Predisposing factors

Tularemia has been reported to occur in any age group in humans. Men tend to have a higher prevalence than women (Mandell and Bennett 2005). However, in Turkey, the number of cases in females is around 1.8 times higher than in males (Kilic 2010). The reason for such greater prevalence in females in Turkey may be due to the more activity of women in household chores. Further, the females may have more contact with contaminated water and with the excreta or urine of animals in areas of food storage (Kilic 2010). People working outdoors and exposed to tick bites such as landscapers, ranchers, forest workers, veterinarians, laboratory workers, and anyone handling the flesh of infected animals have been associated with a higher infection risk (Carvalho et al. 2009). The occurrence of tularemia depends on the type of season, e.g. in spring, summer, and autumn, infections are caused by ticks and mosquito bites while in winter the infections are usually related to drinking contaminated water. In the hunting season, cases related to handling lagomorphs are commonly reported. (CDC USA). *Francisella tularensis* is extremely resistant to environmental stress, surviving for weeks in soil, water, and animal carcasses at low temperatures (Carvalho et al. 2009). Human tularemia outbreaks are often preceded by animal outbreaks, particularly in wild lagomorphs and rodents. This is usually related to an increase in the numbers of these species, increasing the probability of exposure to infected animals (World Health Organization (WHO)) 2007; Carvalho et al. 2009).

The circulation of human infection with *F. tularensis* subspecies *holarctica* has been associated with close vicinity to watercourses where it persists with the protozoa. The recent detection of DNA of *F. tularensis* subspecies *holarctica* in adult mosquitoes, reared from larvae collected in an endemic area, suggests a novel transmission cycle originating in the aquatic habitats of mosquito larvae. However, the dynamics of outbreaks of tularemia remain unknown (Thelaus et al. 2014).

Amebae and main hosts like beavers, muskrats, and lemmings allow *F. tularensis* to persist and spread in aquatic environments. Urine and faeces from sick animals or the carcasses of animals that have died from tularemia are the main causes of water contamination (Bahuaud et al. 2021; Huang et al. 2022). Approximately 500,000 L of water can be contaminated by one diseased water vole or mouse, according to experiments (Sadiku et al. 2022). The bacterium may live in water at infectious levels for more than a month, making it a potentially significant vector for spreading the disease to biting insects, vertebrate animals, and humans (Sadiku et al. 2022). In the last few decades, reports of water-borne tularemia outbreaks and isolated cases have arisen from all corners of the globe. These illnesses pose a serious threat to public safety and military readiness. *Francisella tularensis* can infect humans in many different ways, including by ingestion, as well as through contact with contaminated water during activities like swimming, canyoneering, and fishing (Sullivan et al. 2022). First identified in the 1930s in the Soviet Union, tularemia can also spread through water. Yet, as is customary, it gained steam, and in the last 20 years, numerous countries—including Turkey, Kosovo, Bulgaria, Georgia, Norway, Sweden, Italy, and Germany—have seen a spike in cases connected to drinking water (Hayoun and King 2022). In Turkey, every single human case can be traced back to a water source and is virtually certainly linked to the country’s widespread reliance on bottled water (Kutlu et al. 2021). The water supply was eventually linked to massive outbreaks in Kosovo and Bulgaria, each of which affected hundreds of people. Also, in Georgia, Macedonia, and Norway, tens of human cases traced back to the water supply have been documented (Snowden and Simonsen 2023).

## 9. Prevalence in animals

*Francisella tularensis* infects many vertebrates, but rodents and lagomorphs especially are involved in epizootics. There are no significant figures on the incidence of either naturally occurring infection or disease in these animals. Occasionally, fatal epizootics occur in domestic and wild animals (red fox, *Vulpes vulpes* and beech marten, *Martes foina*), especially rodents and rabbits, causing a dramatic reduction in their numbers (Origgi et al. 2013; Schulze et al. 2016; Pilo 2018). Voles (*Microtus* spp.) exposed *via* oral route may develop chronic bacteriuria (Bell and Stewart 1983). Tularemia in cats has not been reported outside North America which indicates that it is either caused only by *F. tularensis* subspecies *tularensis* or some unknown link has not come to light. Dogs seem to be more resistant to *F. tularensis* and sporadic cases have been reported from North America and Norway (Meinkoth et al. 2004; Nordstoga et al. 2014). *Francisella tularensis* subspecies *holarctica* has been isolated from beavers in North America (Pilo 2018). Birds are also considered resistant to *F. tularensis* infection and infrequent infections have been reported in North America and Sweden (Morner and Addison 2008). A critical role is played by the birds in spreading *F. tularensis* globally. Importantly, in certain regions birds, hares, and rodents are found to carry the same ticks. For this reason, it is thought that they play a role in contributing to the persistence of the bacteria in nature (Sahin 2009; Padeshki et al. 2010). Tularemia in sheep associated with *F. tularensis* subspecie*s tularensis* has been reported in North America (O’Toole et al. 2008). In Iran, *F. tularensis* has been detected in different animals such as porcupines, cattle, and sheep (Fooladfar and Moradi 2023). However, sufficient studies are lacking across different animal species to validate their susceptibility or resistance to *F. tularensis* and its subspecies. Therefore, further investigations are needed to be done on animals (Pilo 2018).

## 10. Prevalence in humans

Tularemia in veterinarians has not been frequently discussed. However, veterinarians have an increased risk for tularemia especially while handling infected dogs and cats. Most of the cat-associated cases (Gliatto et al. 1994; Woods et al. 1998) have been ulcero-glandular secondary to cat bite, with the development of a localized lesion and lymphadenopathy. Cats were not always ill but generally had a history of hunting or wild animal exposure. The contact with the saliva of an ill dog puppy led to an infection in a girl causing typhoidal tularemia. Pulmonary tularemia developed in seven cases exposed to dogs that hunted rabbits (Greene and DeBay 2006). An increase in the number of cases in Bosnia and Kosovo during the conflict suggested that social disruption could also be a risk factor for this disease (Foley and Nieto 2010).

**Figure 3.**
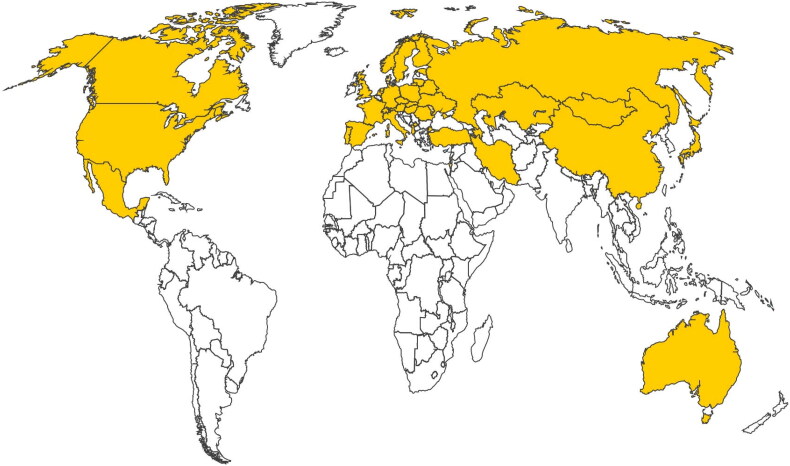
World map of tularemia in humans. Yellow colour shows those countries where tularemia is an autochthonous disease.

Recent outbreaks of tularemia have occurred in several European countries, including the Czech Republic, Kosovo, Bulgaria, Germany, Sweden, Finland, Spain, Turkey, France, Norway, Poland, Belarus and Ukraine. In Europe, the number of cases in humans is approximately 800 per annum. Sweden and Finland are reporting the highest notification rate in the European Union, though tularaemia does not occur in some European countries like Iceland, Ireland, and the United Kingdom (European CDC). A water-borne epidemic of tularemia had been reported in the village of Bursa in the year 2004 wherein children were affected. In this outbreak, the oro-pharyngeal form of the disease was found to be predominant (Celebi et al. 2008). In 2000, an outbreak of tularemia occurred in parts of Sweden where the disease had until then been rare. The affected cases were compared with the controls, and new emerging areas were compared with the endemic areas. The analysis showed that the main risk factors in endemic areas included farming, mosquito bites, and owning a cat. The affected cases showed pneumonia in endemic areas, but swollen lymph nodes and wound infections in emerging areas. The study highlighted the important role of mosquito bites in the transmission of tularemia (Eliasson et al. 2006). The first case of tularemia in Poland was diagnosed in 1949 and since then more than 600 cases have been reported, mainly in the north-eastern and north-western regions of the country. A recent outbreak was recorded in Sweden in 2019 where 979 cases of ulcero-glandular tularemia had been reported following an outbreak in hares (Dryselius et al. 2019). Of late, a case of pulmonary tularemia has been reported in a man with a history of wood chopping in Norway (Kravdal et al. 2021) (Figure 3).

Tularemia is an endemic disease in Turkey; the first outbreak was reported in 1936 in the Trakya region. In the years following this outbreak, many epidemics or sporadic cases were reported from different sites in Turkey (Meric et al. 2010). Re-emergence of tularemia has been reported in Spain due to the persistence of local reservoirs of infection rather than the reintroduction of new strains. The initial report in 1997 affected 559 humans who were connected with the hunting and handling of hares (*Lepus europaeus*). The second major outbreak in the same area in 2007 concurred with a peak in the population of the common vole (*Microtus arvalis*). In between these two major outbreaks, periodic tularemia cases and small outbreaks were reported from 2000–2006 (Ariza-Miguel et al. 2014) (Figure 3).

According to the CDC, the number of human tularemia cases in the United States has decreased over the years (1950-2020). Tularemia has been reported in all the age groups with males being more affected because of a greater probability of contact through hunting and landscaping. An outbreak in South Dakota was associated with very high dog populations and corresponding increases in American dog tick, *Dermacentor variabilis*, and infestation of dogs and human beings (Saliba et al. 1966). A particularly interesting series of cases occurred on Martha’s Vineyard in Massachusetts (Feldman et al. 2001). The risk factors involved were landscaping and two adolescents developed disease apparently by aerosol after running over lagomorphs with a lawnmower. This led to the development of pulmonary tularemia in 15 persons due to *F. tularensis* subspecies *tularensis* (Figure 3). One case each of isolation of *F. tularensis* from blood culture and chest infection of patients in India and Pakistan, respectively has been reported (Nirkhiwale et al. 2015; Ali et al. 2019).

## 11. Manifestations in animals

The clinical signs of tularemia in wild animals are not well-documented, and non-specific post-mortem findings such as splenomegaly and necrotic lesions in the liver and spleen are observed. In France, an outbreak in brown hares revealed splenomegaly, congestion, hemorrhagic lesions in different organs, tracheitis, and bronchitis. A similar study carried out in Hungary on European brown hares naturally infected with *F. tularensis* subspecies *holarctica* also showed very similar results (Carvalho et al. 2014). *Francisella tularensis* can also infect birds, fish, pigs, horses, and hamsters. Infected animals may not show any sign of illness while others may have a fever, inactive, ataxia, depression, refuse to eat, huddle together, ruffle fur, vomiting, and diarrhoea. Further, there may be an increase in pulse as well as respiratory rates, coughing, diarrhoea, and lymphadenopathy along with enlargement of the liver and spleen. Within a few hours to days, prostration and death may occur. Sudden death may also occur in infected animals, especially in rabbits and rodents (CFSPH Technical Fact Sheets XXXX. Tularemia at http://www.cfsph.iastate.edu/DiseaseInfo/; https://www.msdvetmanual.com/generalized-conditions/tularemia/tularemia-in-animals).

Cats usually develop severe illness with unspecific clinical signs like fever, lethargy, prostration, vomiting, anorexia, dehydration, regional or generalized lymphadenopathy, splenomegaly, tongue and oro-pharyngeal ulceration, and jaundice. Pathological findings include multiple necrotic foci on the lymph nodes, spleen, liver, and lungs. Frequently, panleukopenia with toxic degeneration of the neutrophils and hyperbilirubinemia with bilirubinuria are present. Dogs are less susceptible and rarely manifest signs of the disease. Nevertheless, they can act as carriers and transmit the pathogen through their fur after contact with contaminated dead animals or soil. In most cases, infection is self-limiting, and recovery is spontaneous. However, only a few cases of natural infection in dogs have been reported. Infected dogs may have poor appetite, lethargy, and mild fever. Less frequently, they may show conjunctivitis, uveitis, draining abscesses, and enlarged lymph nodes (Williams and Downing XXXX. https://vcahospitals.com). However, a report on tularemia in a dog in Norway was published in 2014. The dog developed clinical manifestations after hunting a mountain hare (*L. timidus*). The case was investigated by serology and a 32-fold increase in antibody titer in two weeks was noticed by the authors. However, the case could not be bacteriologically confirmed, but *F. tularensis* subspecies *holarctica* was isolated from the bone marrow of the captured mountain hare (Nordstoga et al. 2014). The clinical signs in Prairie dogs include lethargy, dehydration, and grossly enlarged cervical lymph nodes. A serological study indicated the potential role of Prairie dogs as reservoirs of *F. tularensis* (Carvalho et al. 2014).

## 12. Manifestations in humans

Depending on the route of transmission, tularemia can develop into different clinical forms (Tarnvik and Berglund 2003; World Health Organization (WHO)) 2007). The incubation period is typically 3 to 5 days but can be up to 20 days. The *F. tularensis* bacterium can parasitize neutrophils besides macrophages and epithelial cells. The clinical signs depend on strain virulence, infective dose, infection route, the extent of systemic involvement, and host immune status. The disease may present in different clinical forms viz., ulcero-glandular, glandular, oculo-glandular, oro-pharyngeal, typhoidal, and pneumonic with the pulmonary and typhoidal forms being the most severe (Dennis et al. 2001; World Health Organization (WHO)) 2007; Sjoested 2007; Yeni et al. 2021). The disease has an acute onset, with the occurrence of fever (38–40 °C), chills, fatigue, generalized myalgia, and headaches, resembling flu. The subspecies *tularensis* (type A) causes severe disease, potentially fatal if untreated. The subspecies *holarctica* (type B) causes milder disease and fatalities are rare (Carvalho et al. 2014; Kelson et al. 2022).

The ulcero-glandular tularemia is characterized by ulcerations in the skin and inflammation of regional tissues and lymph nodes. In glandular form, there are no skin ulcers but lymphadenopathy is marked (Snowden and Simonsen 2023). The oculo-glandular tularemia is an uncommon kind of ulcero-glandular disease in which the conjunctiva becomes infected first, typically due to the transmission of bacteria on the fingertips. Ulcers and nodules form on the conjunctiva, and the infection spreads to the regional lymph nodes if left untreated (Yeni et al. 2021; Bishop et al. 2023). In oro-pharyngeal tularemia, a painful sore throat, enlarged tonsils, and the development of a yellow-white pseudomembrane are common manifestations. The cervical lymph nodes tend to swell in tandem with this condition. It may lead to mild but chronic diarrhoea or a life-threatening illness marked by severe ulceration of the colon (Boeckel et al. 2022; Rijks et al. 2022; Sullivan et al. 2022). Typhoidal tularemia can be caused by type A or type B strains, although usually less severe for type B. It is a systemic disease usually accompanied by neurological symptoms, but without a detectable portal of entry of bacteria (e.g. absence of skin ulcer) and without regional infection. Pneumonic tularemia can manifest mainly as pneumonic lesions, pleurisy, and media­stinal or hilar lymphadenopathy (Snowden and Simonsen 2023).

In humans, besides the clinical forms that are well characterized, tularemia may also be responsible for causing secondary pleuropneumonia, meningitis, and sepsis ultimately leading to shock and subsequent death (American Veterinary Medical Association 2003). The mortality rates vary greatly depending on the *F. tularensis* strain type (A or B), the clinical form, the patient’s underlying health condition, the precocity of adequate treatment, etc.

## 13. Pathogenesis

**Figure 4.**
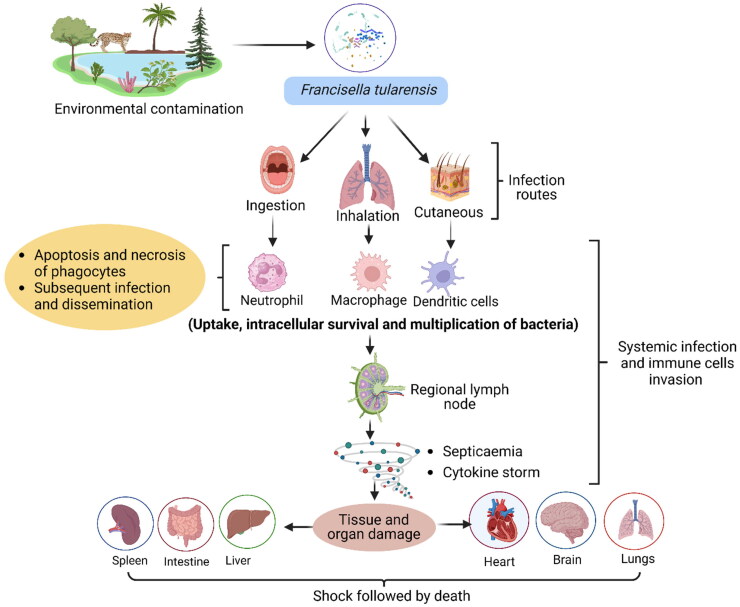
Pathogenesis of tularemia.

*Francisella tularensis* infects a variety of host-cell types, including macrophages, dendritic cells, neutrophils, hepatocytes, endothelial, and type II lung epithelial cells (Celli and Zahrt 2013). After phagocytosis, the bacterium escapes quickly from the phagosome into the cytoplasm where they replicate leading to apoptotic death of the host cell, bacterial release, and subsequent infection and dissemination (Celli and Zahrt 2013). It evades the immune responses and can replicate to high levels in the liver, spleen, and lungs before the immune system is provoked to respond with a destructive cytokine storm (Ramakrishnan 2017; Singh et al. 2017) (Figure 4).

Acute pulmonary infection with *F. tularensis* in mice elicits a marked response of the myeloid cells characterized by the migration of a large number of immature myeloid cells and myeloid-derived suppressor cells to the lungs. These myeloid cells do not mature and later die, leading to necrosis in the lungs followed by multi-organ failure and death. Interleukin (IL)-1β favours the maturation and differentiation of more-effective phagocytic cells over IL-1α during acute pulmonary infection (Periasamy and Harton 2018). However, the Nlrp3 inflammasome promotes acute necrotic pulmonary tularemia by limiting the infiltration of protective mature neutrophils (Periasamy et al. 2016b). Conversely, during sub-acute infection, the host response is significantly altered with migration and the development of mature myeloid cells that are more effective at controlling *F. tularensis* (Periasamy et al. 2016a).

*Francisella* resists reactive oxygen species (ROS) and products in the phagosome including superoxide (O_2_^−^) anions, hydrogen peroxide (H_2_O_2_), hypochlorous acid (bleach), and peroxynitrite (ONOO^−^) by enhancing the production of antioxidant enzymes like superoxide dismutase, catalase, and alkyl hydroperoxide reductase. These antioxidant enzymes are regulated by the oxidative stress response regulator (OxyR). Thioredoxin (TrxA1) of *F. tularensis* plays a major role in the oxidative stress response of *F. tularensis* by regulating the expression of the master regulator, *oxyR* (Ma et al. 2019). Besides, it was found that TrxA1 is vital for the intramacrophage survival and growth of *F. tularensis* (Ma et al. 2022). Differences in the antioxidant defense mechanisms of the *F. tularensis* live vaccine strain (LVS) and the highly virulent SchuS4 strain and their abilities to counter oxidative and nitrosative stresses have been reported (Alharbi et al. 2019). Alkyl hydroperoxide reductase C (AhpC), a peroxiredoxin of *F. tularensis* confers resistance against a wide range of ROS and reactive nitrogen species and serves as a virulence factor. The AhpC functions as the main antioxidant enzyme and contributes to the oxidative and nitrosative stress resistance, and intramacrophage survival of the highly virulent SchuS4 strain (Alharbi et al. 2019). *Francisella tularensis*-induced inhibition of NADPH oxidase assembly depends on opsonization. It is observed with strains from both *F. tularensis* subspecies *holarctica* as well as *tularensis*. A unique ability is possessed by *F. tularensis* for inhibiting the activity of NADPH oxidase (post-complex assembly). This event limits the activation of neutrophils by other stimuli subsequently. Ultimately, the production of ROS is diminished following the phagocytosis of the antibody-opsonized bacterium (McCaffrey et al. 2010; Geier and Celli 2011).

One of the recent studies highlighted the damaging role of Nlrp3 in causing respiratory tularemia in mice. It was found that Nlrp3 suppressed the response of pro-inflammatory cytokines in *F. tularensis* infection. The levels of IL-1β, TNF-α, other pro-inflammatory cytokines, and chemokines were found to be increased in response to loss of Nlrp3 in *F. tularensis*-infected macrophages. Furthermore, the Nlrp3-negative macrophages and mice were able to clear the bacteria more efficiently than their corresponding wild-type counterparts. These findings established that Nlrp3 could be a prospective target for the development of effective therapeutics for tularemia (Suresh et al. 2021).

## 14. Diagnostic procedures

Diagnosis of tularemia can be achieved by adopting various methods. However, tularemia in rabbits is usually diagnosed only at post-mortem based on appropriate, non-specific gross and microscopic lesions and the isolation of *F. tularensis*. In humans, the diagnosis of tularemia is difficult also due to the presentation of non-specific symptoms. Since the disease is more common during summer, clinicians should suspect the condition during this season (Kelson et al. 2022).

### 14.1. Samples

In humans, samples should preferably be collected before the onset of anti-biotherapy and the sample type depends on the clinical form of the disease. Samples may include non-heparinized whole blood, serum, secretions and washes from the respiratory tract, swabs from discernible lesions, aspirates or biopsies from lymph nodes, urine, and autopsy materials. Lymph nodes or bone marrow aspirates, organs (lungs, liver, spleen), and cerebrospinal fluid can also be used. Blood samples for antibody tests should be collected at least 14 days after the start of the symptoms. In the context of an outbreak or epidemiologic studies, samples should include arthropod vectors as well as environmental samples like water, soil, and rodent faeces (World Health Organization (WHO)) 2007; Yeni et al. 2021; Kelson et al. 2022; Fooladfar and Moradi 2023).

### 14.2. Culture

Culture is the gold standard for diagnosis of *F. tularensis* infection and must be carried out in biosecurity level 3 (BSL-3) facilities. *Francisella tularensis* is a fastidious microorganism and is isolated in less than 10% of tularemia patients. Optimal growth conditions occur at 37 °C and pH 6.9. Cysteine-enriched media, such as enriched chocolate agar (CA) or 9% cysteine heart agar with blood medium (CHAB) must be used for this purpose. Growth in a CHAB medium enables the presumptive identification of *F. tularensis* by characteristic growth at 24-48 h of round and smooth green opalescent shiny colonies, 2–4 mm in diameter (World Health Organization (WHO)) 2007).

### 14.3. Microbiologic identification of F. tularensis

The microbiological identification of *F. tularensis* can be obtained by MALDI-TOF mass spectrometry provided that an appropriate spectrum database is used. It is a tool for accurate and quick identification and typing of *F. tularensis* strains (López-Ramos et al. 2020; Regoui et al. 2020). However, a definite species and subspecies identification now requires molecular methods. Furthermore, genome sequencing is needed to find out the genotype, which is useful for determining the probable geographic origin and virulence of a given strain (Wagner et al. 2022). Biochemical identification of *F. tularensis* can no longer be considered as reliable.

### 14.4. Serology

A seroconversion or a fourfold rise in antibody titers between acute and convalescent phase sera are diagnostic confirmations of tularemia. A significant antibody titer on a single serum sample is only considered a probable tularemia case. Serologic methods include the whole-cell agglutination test (Widal’s reaction), the tube agglutination test, microagglutination assays, hemagglutination, enzyme-linked immunosorbent assay (ELISA), and immunoblot. The commercialized ELISA tests are highly sensitive but lack specificity. Most tularemia cases are diagnosed by serology tests. The main difficulty is the long-term persistence of specific antibodies in some tularemia patients. Therefore, it is sometimes challenging to differentiate past from recent infections with *F. tularensis*.

The same approach can be used for animals. Serology has limited use in highly susceptible species since death usually precedes the development of specific antibodies. However, in endemic areas, antibodies for *F. tularensis* are frequently detected in wild animals that have developed immunity, including foxes and coyotes. This seroconversion is suspected of being related to subspecies *holarctica* infection since infection by the subspecies *tularensis* is expected to be fatal (Mandell and Bennett 2005; World Health Organization (WHO)) 2007). A latex agglutination test (LAT) has also been developed for quickly identifying the subspecies of *F. tularensis* that are clinically relevant in the case of humans. Within three minutes, the test can be done with either live or inactivated bacteria. The risk of contracting infection in the laboratory is minimized by the use of inactivated samples and thus the test can be performed under a biosafety level 2 (BSL-2) environment (Rastawicki et al. 2018). Maurin (2020) recommended the use of ELISA along with a microagglutination test (MAT) and indirect immunofluorescence assays (IFA) for serological diagnosis of tularemia. It has been found in his study that specific antibodies can be detected by ELISA within 14 days of evaluation of disease in comparison to 2-3 weeks for IFA and MAT. Further serological cross-reactions have been observed in tularemia with *Brucella* as well as *Yersinia* species. According to Maurin (2020), such cross-reactions can be highlighted by the immunoblotting technique.

### 14.5. Molecular methods

Molecular methods are valuable diagnostic tools when compared to conventional methods. Many real-time PCR tests are now available to detect *F. tularensis* in clinical samples, some being species-specific, and others subspecies or even genotype-specific (Versage et al. 2003; Bystrom et al. 2005; Splettstoesser et al. 2005; World Health Organization (WHO)) 2007; Vogler et al. 2009; Simşek et al. 2012; Larson et al. 2020; Rahravani et al. 2022). Further characterization of the involved *F. tularensis* strain is now usually done by genome sequencing (Champion et al. 2009; Wagner et al. 2022).

Additional discrimination has been achieved using high-resolution genotyping methods including pulse-field gel electrophoresis (PFGE), whole-genome single-nucleotide polymorphism (SNP) phylogeny, amplified fragment-length polymorphism (AFLP), ribotyping, 16S rDNA gene sequencing, canonical insertion deletions and paired-end sequence mapping (Fey et al. 2007; Larsson et al. 2007; Johansson et al. 2014). Microarrays have also allowed for the differentiation of the *F. tularensis* subspecies and have been proven useful for pathogenicity and virulence marker identification. One area of important recent research is the application of highly sensitive molecular techniques such as variable number tandem repeats (VNTR) to confirm point sources of outbreaks and relatedness of geographically and temporally proximal cases (Foley and Nieto 2010).

## 15. Animal disease management

Treatment of tularemia in rabbits is not recorded since antemortem diagnosis is rarely made and wildlife populations are not available for treatment. Streptomycin and tetracycline are the antibiotics of choice. Though gentamicin and chloramphenicol, are also effective. Gentamicin should be administered for 10 days. Since tetracycline and chloramphenicol are bacteriostatic, they should be administered for 14 days to curtail the risk of relapse. Early treatment should prevent death loss. Prolonged treatment may be necessary because many organisms are intracellular (CDC USA website 2023; www.avma.org).

## 16. Human disease management

Antibiotic treatment of human tularemia is based on aminoglycosides (gentamicin and streptomycin), tetracyclines (mainly doxycycline), and fluoroquinolones (ciprofloxacin, but also levofloxacin and moxifloxacin) (Yeni et al. 2021; Kelson et al. 2022). It is pertinent to mention that streptomycin is no longer available in many countries. Tigecycline should not be used for treating tularemia because it has too large an antimicrobial spectrum and would therefore unnecessarily destroy the patient’s microbiota. Many patients with lymphadenopathy evolve to lymph node suppuration often requiring surgical drainage or excision (Gozel et al. 2014; Ozkaya-Parlakay and Polat 2021).

## 17. Prevention and control strategies

It is very challenging to control the disease in wildlife. However, wild rabbits and hares should be handled with great caution in areas where tularemia is endemic. The control of the disease in man can be effective to some extent. Professional workers should be educated about the disease and advised to wear rubber gloves and masks when skinning and dressing lagomorphs, avoid ticks and flies in enzootic areas, cook meat thoroughly, and avoid the consumption of non-potable water. Protective clothing and insect repellents should be used to prevent the bite of arthropods. Eradication of rodent populations in limited areas may be effective in reducing the spread of the disease and contact with other animals and man. Control is difficult and is limited to reducing tick infestation and to rapid diagnosis and treatment. Recovery confers long-lasting immunity.

Efforts are currently underway to develop a safe and effective vaccine. Various vaccines against tularemia were developed in the 1900s that included the killed ‘Foshay’ vaccine, and subunit vaccines that comprised proteins or lipoproteins of the organism in an adjuvant formulation. However, none of these vaccines were given licenses in the United States or European Union (Jia and Horwitz 2018). Currently, there is no available licensed vaccine against *F. tularensis* although an attenuated type B strain, known as the Live Vaccine Strain (LVS) was developed in the United States during the 1950s and used to vaccinate military personnel and laboratory workers. The LVS vaccine failed to uniformly protect against pneumonic tularemia and when delivered in high titers caused mild tularemia as an undesirable side-effect. One focus of current research work in the United States and Europe is to develop a vaccine for protection against *F. tularensis* intentional release (World Health Organization (WHO)) 2007). The restricted efficacy of the LVS has fostered extensive research intending to provide alternative vaccine formulations, including the exploration of different live and killed attenuated strains and immunogenic components to produce subunit vaccines. Given its immunogenic antigens, an effort has been made to develop attenuated strains of SchuS4, a representative strain of type A *F. tularensis*, as a vaccine component. In fact, between LVS and SchuS4 strains, there are about 35 genes that encode for different protein sequences, whose functions are not well-defined, and may represent important immunogens. Still, given the increased virulence of the SchuS4 strain, only a small number of bacteria should be required to generate effective protection against wild-type A *F. tularensis* (Rockx-Brouwer et al. 2012). Investigations have been carried out to know the potential of outer membrane proteins (OMPs) of *F. tularensis* as a subunit (acellular) vaccine. Protection was provided in 50% of the mice against challenge with SchuS4 by immunizing with three doses of adjuvanted native OMPs (Huntley et al. 2008; Barry et al. 2009).

A tetravalent subunit vaccine developed by conjugating OmpA, DnaK, Tul4, and SucB proteins of *F. tularensis* to the Tobacco Mosaic Virus and CpG as the adjuvant for respiratory tularemia provided 100% protection against a 10 LD_100_ intranasal challenge dose of *F. tularensis* in mice by inducing strong protective and memory immune responses (Banik et al. 2015; Mansour et al. 2017).

## 18. Conclusion and future directions

Numerous aspects such as human demographics and behaviour, international travel, and commerce, including the animal trade, climatic changes, and microbial adaptations, have a potential impact on disease ecology and the emergence of zoonosis. The same factors are thought to be related to the emergence of tularemia. The major concern is that the *F. tularensis* can act as a potential bioterrorism weapon by its high infectivity rate, and easy spread through aerosols and contaminated water. Besides, tularemia is reported from wide geographical areas with the emergence of new regions, mainly in Europe. There is a need to increase awareness of the disease among risk populations, particularly hunters and health professionals. The advanced research will help to identify and characterize new circulating strains of *F. tularensis* and develop molecular and typing methods with augmented sensitivity and specificity. It is also pertinent to remember that *F. tularensis* can infect a wide range of hosts and vectors than most other zoonotic pathogens, though the exact mechanism of adaptation to many arthropod vectors is not well understood.

Despite our increasing knowledge of tularemia and its etiological agent, many aspects of *F. tularensis* biology and epidemiology need to be further examined, particularly its pathogenicity and virulence, vaccine development, and the specific mechanisms by which *F. tularensis* evades, modulates, and suppresses the host immune response. More information is needed about the wild and domestic animals which may act as reservoirs of *F. tularensis*. Since the disease pattern in humans is linked with the increase in the population of lagomorphs and rodents in the area, therefore these factors should also be included in the surveillance programs. From a public health perspective, disease surveillance in animals is crucial to prevent and monitor human outbreaks, particularly in endemic areas, where contact between humans and wildlife reservoirs or a vector is likely. The medical and veterinary health system should be future prepared to deal with the impact of any impending major tularemia zoonosis.

Humans can contract tularemia from a wide variety of vector bites, water contact, aerosol exposure, and the handling or eating of infected animal materials, all of which contribute to the disease’s distinctive characteristics. There may be significant differences between the environmental conditions the bacterium has evolved and those of another site. Tularemia has been likened to a chameleon because of its ability to change its appearance in response to its surroundings. Tularemia can spread easily between animals and from animal to human in certain climates. Ticks are abundant, and the pathogen is common, therefore conditions are ideal for the spread of this disease. Furthermore, migratory birds pose a persistent risk for the introduction of novel illnesses in several areas. Therefore, each country needs its disease management system to keep contagious diseases under control. Public education about the need to take preventative measures against tularemia through the lens of the One Health approach is also important. Controlling tularemia through the creation of reliable vaccines and anti-*Francisella* medications should be a top research priority, and this may be accomplished with the help of experts who can plan and implement cutting-edge research initiatives.
